# In Vitro Compression Model for Orthodontic Tooth Movement Modulates Human Periodontal Ligament Fibroblast Proliferation, Apoptosis and Cell Cycle

**DOI:** 10.3390/biom11070932

**Published:** 2021-06-23

**Authors:** Julia Brockhaus, Rogerio B. Craveiro, Irma Azraq, Christian Niederau, Sarah K. Schröder, Ralf Weiskirchen, Joachim Jankowski, Michael Wolf

**Affiliations:** 1 Department of Orthodontics, Dental Clinic, University Hospital RWTH Aachen, 52074 Aachen, Germany; jbrockhaus@ukaachen.de (J.B.); iazraq@ukaachen.de (I.A.); cniederau@ukaachen.de (C.N.); michwolf@ukaachen.de (M.W.); 2 Institute of Molecular Pathobiochemistry, Experimental Gene Therapy and Clinical Chemistry, University Hospital RWTH Aachen, 52074 Aachen, Germany; saschroeder@ukaachen.de (S.K.S.); rweiskirchen@ukaachen.de (R.W.); 3 Institute for Molecular Cardiovascular Research, University Hospital RWTH Aachen, 52074 Aachen, Germany; jjankowski@ukaachen.de

**Keywords:** mechanical compression, orthodontic tooth movement, human Periodontal Ligament Fibroblast, proliferation, cell cycle

## Abstract

Human Periodontal Ligament Fibroblasts (hPDLF), as part of the periodontal apparatus, modulate inflammation, regeneration and bone remodeling. Interferences are clinically manifested as attachment loss, tooth loosening and root resorption. During orthodontic tooth movement (OTM), remodeling and adaptation of the periodontium is required in order to enable tooth movement. hPDLF involvement in the early phase-OTM compression side was investigated for a 72-h period through a well-studied in vitro model. Changes in the morphology, cell proliferation and cell death were analyzed. Specific markers of the cell cycle were investigated by RT-qPCR and Western blot. The study showed that the morphology of hPDLF changes towards more unstructured, unsorted filaments under mechanical compression. The total cell numbers were significantly reduced with a higher cell death rate over the whole observation period. hPDLF started to recover to pretreatment conditions after 48 h. Furthermore, key molecules involved in the cell cycle were significantly reduced under compressive force at the gene expression and protein levels. These findings revealed important information for a better understanding of the preservation and remodeling processes within the periodontium through Periodontal Ligament Fibroblasts during orthodontic tooth movement. OTM initially decelerates the hPDLF cell cycle and proliferation. After adapting to environmental changes, human Periodontal Ligament Fibroblasts can regain homeostasis of the periodontium, affecting its reorganization.

## 1. Introduction

The periodontium is the connective tissue of the tooth, consisting of the gingiva, cementum, alveolar bone and periodontal ligament. Periodontal Ligament Fibroblasts (PDLF) build the connective tissue between the cementum and the alveolar bone. Interstitially collagenous Sharpey fibers, inserted in the acellular fibrillar cementum, anchor the tooth as a gomphosis in the alveolar cavity [1,2,3].

The aim of orthodontic treatment is the alignment of displaced teeth, eliminating malocclusions and inadequate loading of the periodontium. The side effects of orthodontic tooth movement (OTM) on the periodontium, e.g., root resorption and excessive loosening of the teeth, need to be reduced [4,5,6]. During OTM, the tooth root is divided into a compression and a tension side, perpendicular to the vector of force application [7]. On the tension side, the periodontal ligament is stretched inducing bone apposition and alignment of the Sharpey fibers. In contrast, resorption of the alveolar bone and degradation of the periodontal ligament is observed on the compression side [8]. Clinically and histologically, OTM at the compression side is divided into three phases according to the morphological changes. The first phase is the initial damping, where the tooth is deflected within the alveolar cavity by the width of the periodontal gap, compressing the periodontal ligament. It is followed by a second phase (i.e., hyalinization phase) in which nearly no tooth movement is measurable. The third phase (i.e., resorption phase) is reached when an accelerated and homogeneous tooth movement can be recognized due to direct bone resorption [9].

Within the periodontal ligament, Periodontal Ligament Fibroblasts build the surrounding tissue around the tooth root. They have a broad plasticity to maintain the homeostasis within the periodontium [10,11]. PDLFs are significantly involved in regulating the adaptation of the periodontal apparatus to extrinsic and intrinsic stimuli. Mechanical stress, such as mastication or orthodontic tooth movement and hypoxia, trigger the PDLF cellular response [11,12]. This stimulus is detected through mechanosensitive receptors, ion channels and mechano-transduction and induces the restructuration of the periodontium [13]. The response to mechanical stimulation is mainly initiated through the PDLF activating biochemical signaling pathways to regulate bone remodeling and tissue regeneration. Tissue reorganization through the PDLF response is guided by inflammatory signaling cascades releasing different proinflammatory cytokines and cell death mediators. It has been shown that interleukin-6 and -8 (IL-6 and IL-8), as well as cyclooxygenase 2 (COX-2), tumor necrosis factor-α (TNF-α) and Toll-like receptor (TLR) ligands, have an essential part [14,15,16,17]. These proinflammatory cytokines and cell death mediators are known to play a crucial role during early phase OTM sterile inflammation and other inflammatory reactions such as gingivitis and periodontitis [18]. However, the PDLF function during OTM is not completely understood yet. Therefore, more studies are required to examine the processes within PDLFs that are involved in adaptation and restructuration of the PDL during mechanical stress.

The exact magnitudes of the orthodontic forces on periodontal cells in vivo are variable due to the patient and treatment characteristics [19]. The general issue of OTM is that the applied force should achieve a cellular response without causing excessive tissue damage. Nevertheless, a very well-established in vitro model of the early phase OTM compression side in human Periodontal Ligament Fibroblasts (hPDLF) is already able to induce a reactive synthesis of proinflammatory cytokines and remodeling molecules [20,21]. This model with glass cylinders exerting a static compressive force of 2 g/cm^2^ on a cell monolayer was used to dissect the process involved in the restructuration of hPDLFs.

As mentioned above, inflammation is increased in this compressive force model. This in vitro static compressive force model was used in this study in order to investigate whether there is an influence on the proliferation and cell cycle in hPDLF under mechanical stimulation. The results show that the proliferation rate of hPDLFs correlates with their viability and is regulated by the cell cycle. As proliferation maintains the growth and homeostasis of tissues and correlates with rising inflammatory signaling, the main area of interest is the regulation of the cell cycle through the modulation of the cyclins and cyclin-dependent kinases (CDKs), DNA replication licensing factors (MCM2) and proliferating cell nuclear antigen (PCNA) during the compressive force [22]. Furthermore, the influence of OTM on the cell count over time and morphological changes as potential symptoms of adaptation were examined.

In this study, it was displayed that hPDLFs restructure and adapt to the compressive force. By using a well-established in vitro model for mechanical stress, simulating the first phase of OTM, it was revealed for the first time that cell proliferation is reduced via cell cycle regulation.

It is hypothesized that hPDLFs generally die through mechanical compression and mainly contribute to tissue reorganization of the periodontium during OTM through the inflammatory response. In this in vitro model, hPDLFs restore homeostasis, preserving the cellular structures under static compression through a lower cell division rate and a slowed cell cycle. Based on the results, the reduction of proliferation is indicated to be—parallel to the cellular inflammation and cell death—a crucial part of the reorganization process of hPDLFs under a compressive force.

## 2. Materials and Methods

### 2.1. Reagents and Antibodies

The primary antibodies MCM2 (D7G11 XP^®^, #3619; Cell Signaling Technology, Danvers, MA, USA), PCNA (6D645, #sc-71858; Santa Cruz Biotechnology, Santa Cruz, CA, USA), Cyclin A (H-432, #sc-751; Santa Cruz, CA, USA), LCN2 (AF1757; R&D Systems, Minneapolis, MN, USA), β-Actin (AC-15, #A5441; Sigma Aldrich, Taufkirchen, Germany) and Vinculin (#sc-73614; Santa Cruz, CA, USA) were used. Secondary antibodies conjugated to horseradish peroxidase (mouse anti-goat #31400, goat anti-mouse #31430 and goat anti-rabbit #31460) were purchased from Invitrogen (Waltham, MA, USA). For Western blotting, cells were harvested with RIPA buffer (20-mM Tris-HCl (pH 7.2), 150-mM NaCl, 2% (*w/v*) NP-40, 0.1% (*w/v*) SDS and 0.5% (*w/v*) sodium deoxycholate) supplemented with the Complete^TM^ mixture of proteinase inhibitors (Roche Diagnostics, Mannheim, Germany). For cell cytometry, carboxyfluorescein-succinimidyl ester (CFSE) was purchased from Invitrogen (Waltham, MA, USA), while propidium iodide solution (PI) was purchased from Miltenyi Biotech (Bergisch Gladbach, Germany) and the Annexin V–7AAD Apoptosis assay (PE Annexin V Apoptosis Detection Kit I) by BD Biosciences (Franklin Lakes, NJ, USA).

### 2.2. Cell Culture and In Vitro Compressive Stimulation Model

The hPDLFs were purchased from Lonza Group (Basel, Switzerland) and cultured in high-glucose Dulbecco’s Modified Eagle’s Medium (DMEM; Gibco, Gaithersburg, MD, USA), containing 100 units/mL of penicillin, 100 µg/mL of streptomycin (Gibco, Gaithersburg, MD USA), 10% FCS (Gibco, Gaithersburg, MD, USA) and 50-mg/L L-ascorbic acid (Sigma-Aldrich, St. Louis, MO, USA) at humidified 37 °C and 5% CO_2_. Cells were trypsinized and centrifuged at 350× *g* and then seeded in 6-well plates and let grown to 90% confluence. If required, cells were stained before plating. After a 24-h incubation, a static force of 2 g/cm^2^ (0.02 N/cm^2^, respectively) was applied on the monolayer with sterile round-glass cylinders (34 mm Ø; 18 g), as described and established by Kanzaki et al. [21]. At the same time, the first samples without a compressive force were harvested, marking the 0-h time point. Following the recommendations of Janjic et al., the durations of mechanical compression were 24 h, 48 h and 72 h [23].

### 2.3. Trypan Blue Staining—Quantification of Cell Numbers

At 24 h, 48 h and 72 h, the glass cylinder was removed, and the supernatant from both CF and controls (0 h–72 h) was collected in a 15-mL Falcon tube. The cells were rinsed with phosphate-buffered saline (PBS; Gibco, Gaithersburg, MD USA) and detached with 200-µL Trypsin and harvested with 2-mL PBS in the same Falcon tube, centrifuged for 5 min at 350× *g* at room temperature and resuspended in 200-µL PBS. The labeling of dead cells was performed by using 0.4% trypan blue staining solution (Gibco, Gaithersburg, MD, USA), according to the manufacturer’s specifications. Quantification was performed in a Neubauer counting chamber. All experiments, including the identification and counting of living cells and blue-stained dead cells, were carried out by the same operator.

### 2.4. Phalloidin/DAPI Staining

To visually compare cells with and without mechanical loading compression, the cells were fixated in 3.7% formaldehyde suspension (Carl Roth, Karlsruhe, Germany). Afterwards, the cells were permeabilized in a PBS supplemented with 0.1% Triton X-100. Subsequently, samples were incubated and blocked in PBS containing 1% BSA (Carl Roth, Karlsruhe, Germany), followed by a 1× rhodamine phalloidin stain (Thermo Fisher Scientific, Waltham, MA, USA) and an antifade solution containing 4′,6-diamidino-2-phenylindole (DAPI; Molecular Probes, Thermo Fisher Scientific, Waltham, MA, USA), according to the manufacturer’s protocol. Subsequently, the monolayer was covered for preservation with coverslips and examined by immunofluorescence imaging microscopy (DMi8 fluorescence microscope; Leica, Wetzlar, Germany).

### 2.5. Combined Cell Proliferation and Apoptosis Assay

Human PDLFs were stained with CFSE, according to the supplier’s instructions, and plated in 6-well culture dishes. Thereafter, both floating and attached cells were collected and stained with PI solution (Miltenyi Biotech, Bergisch Gladbach, Germany) and analyzed by flow cytometry in a BD FACS Canto II (BD Biosciences, San Jose, CA, USA). Cell death was detected by combined 7AAD/Annexin V-antibody, according to the manufacturer’s instructions.

### 2.6. Isolation of RNA, Purification and cDNA Synthesis

Two milliliters of PBS were used to rinse the monolayer. Then, 0.5-mL TRIzol™ Reagent (Thermo Fisher Scientific, Waltham, MA, USA) were applied to harvest the cells. After RNA isolation, the Qiagen RNeasy Mini Kit (Qiagen, Hilden, Germany) combined with an on-column DNA digestion (RNase-Free DNase, Qiagen, Hilden, Germany) was used for RNA purification according to the manufacturer’s instructions. The RNA concentration and chemical purity of each sample was determined photometrically at 280-nm and 260-nm by Nanodrop One™ (Thermo Fisher Scientific, Waltham, MA, USA). Five micrograms of each sample were reversely transcribed into the cDNA using SuperScript III RT (Thermo Fisher Scientific, Waltham, MA, USA) at 50 °C for 60 min, followed by 70 °C for 15 min.

### 2.7. Real-Time Quantitative PCR

For real-time (RT) qPCR, the intron-spanning primers (Eurofins, Luxembourg, Luxembourg) were designed by using Primer-BLAST (NCBI) and the Roche Universal Primer Library Tool (Roche Diagnostics, Mannheim, Germany), as mentioned in our work by Niederau et al. [24]. In silico qPCR specificity was examined with a PCR check (Eurofins Oligo Analysis Tool, Luxembourg) regarding intron-spanning, length (20 bp) and product length (200 bp), transcript variations and annealing temperature. Each cDNA sample was analyzed in technical duplicates using 2.5-ng/µL cDNA with a primer concentration of 0.5-µM and High Green Mastermix (Thermo Fisher Scientific, Waltham, MA, USA). Real-time (RT) qPCR was carried out with qTower3 and qPCR-Soft 3 (Analytik Jena GmbH, Jena, Germany), with a 2-min initial warming (50 °C) and heating up to 95 °C for 10 min, followed by 40 cycles of 95 °C/15 s, 60 °C/30 s and 72 °C/30 s. All primers are displayed in Table 1. Additional primer information about the gene function, acc. no., chromosomal location and primer location, amplicon length and location can be found in the Supplementary Materials Table S1.

### 2.8. Western Blot Analysis

For the protein analysis, hPDLFs were harvested in 100-µL/well RIPA buffer, as mentioned above. The epithelial human prostate cell line PC-3 (human adenocarcinoma cells; PC-3; ATCC^®^ CRL-1435™), originally derived from a 62-year-old patient, was used as a positive control, as it showed a high expression for all the proteins of interest [25]. The cell line was obtained from LGC Standards GmbH (Wesel, Germany) and routinely cultured in DMEM supplemented with 10% FCS, 100-U/mL penicillin, 100-μg/mL streptomycin and 2-mM L-glutamine. A Western blot analysis was essentially performed as previously described in Schröder et al. [26]. Briefly, protein amounts were determined by the DC protein assay (Bio-Rad Laboratories GmbH, Düsseldorf, Germany). Equal amounts of protein (hPDLF: 20-µg; PC-3: 5-µg) were loaded onto 4–12% Bis-Tris gradient gels. After fractionation, the samples were electroblotted on a 0.45-µm nitrocellulose membrane. Equal protein loading and successful transfer was demonstrated by Ponceau S staining. After blocking (one hour at room temperature) in TBS-T (TRIS-buffered saline and 0.1% Tween-20) supplemented with 5% nonfat milk powder, the membranes were incubated with primary antibodies diluted according to the manufacturer’s instructions. To show equal protein loading, β-Actin was stained as an endogenous control. Final visualization of the proteins was conducted with horseradish peroxidase-linked secondary antibodies (anti-mouse, anti-rabbit or anti-goat IgG) and the SuperSignal chemiluminescent substrate (Thermo Fisher Scientific, Waltham, MA, USA). The Western blot raw data can be found in the Supplementary Materials Figure S1.

### 2.9. Statistical Analysis

**Figure 1:**
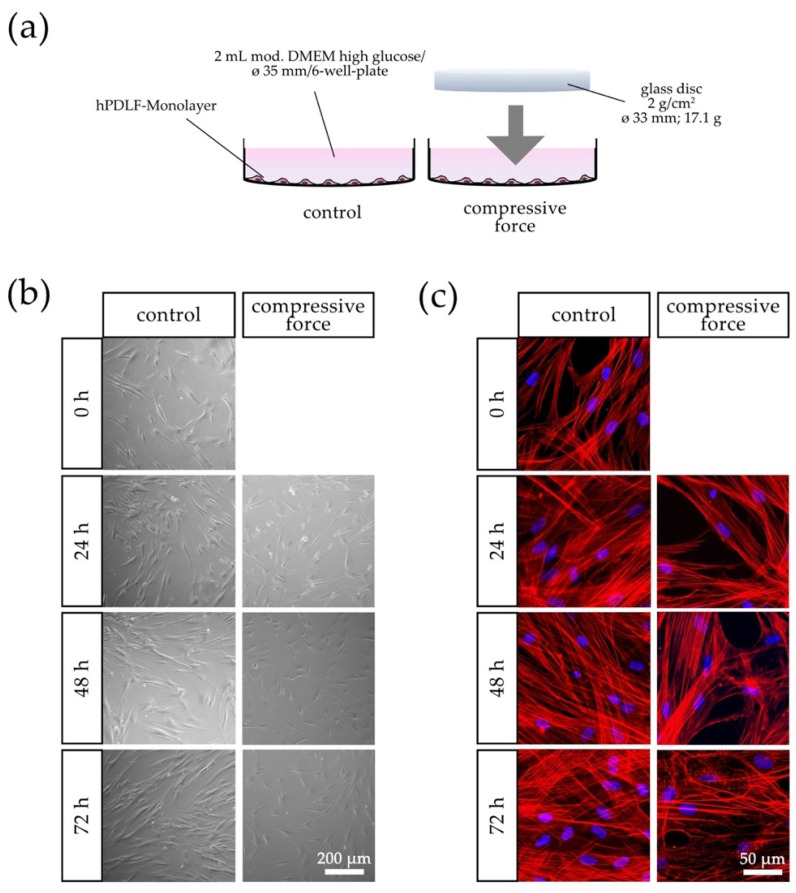
Effects of compressive force (CF) on hPDLFs in terms of confluence and morphology compared to the control group at 0–72 h. (**a**) Schematic representation of the CF in vitro model with a sterile glass cylinder placed on the monolayer. (**b**) Light microscopic and (**c**) fluorescence microscopic phalloidin/DAPI photographs. In addition to the confluence reduction at each time point, morphological changes were especially evident after 72 h in the CF group due to the disorganization of actin filaments and punctuate accumulation of aggregates.

**Figure 2:**
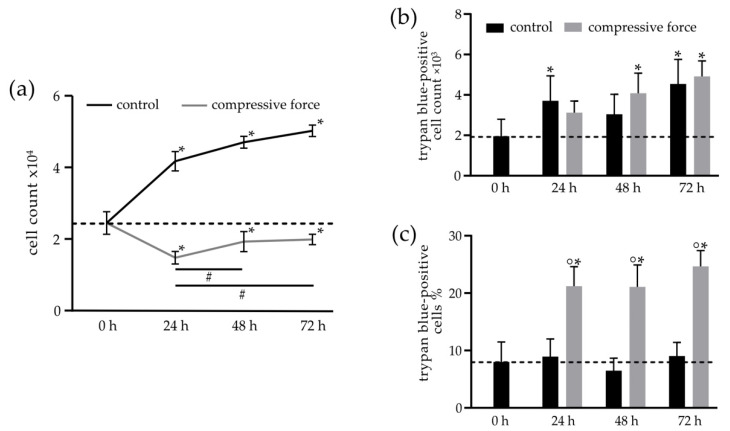
Influence of the compressive force (CF) on the cell count and cell death rate, examined by trypan blue staining. (**a**) Quantification of the total cell numbers ×10^4^ in the control (C) and CF groups at 0–72 h. After 24 h, there was a significant reduction in cell numbers compared to the 0-h control in the CF group. Subsequently, the population recovered and approached the baseline value of 0 h again. (**b**) The total number of trypan blue-positive cells increased significantly in both the C and CF groups compared to the 0-h time point. (**c**) A higher percentage of trypan blue-positive cells over the entire study period and unchanged percentage of dead cells in the C group. Bars indicate the mean values  ±  standard deviations. A *p*-value of *p* < 0.05 was considered statistically significant. (*) Comparison of the 24-h, 48-h and 72-h (C and CF) time points with the 0-h control. (°) Comparison of the CF with the respective controls of the time points. (#) Comparisons between different compressive force time points.

**Figure 3:**
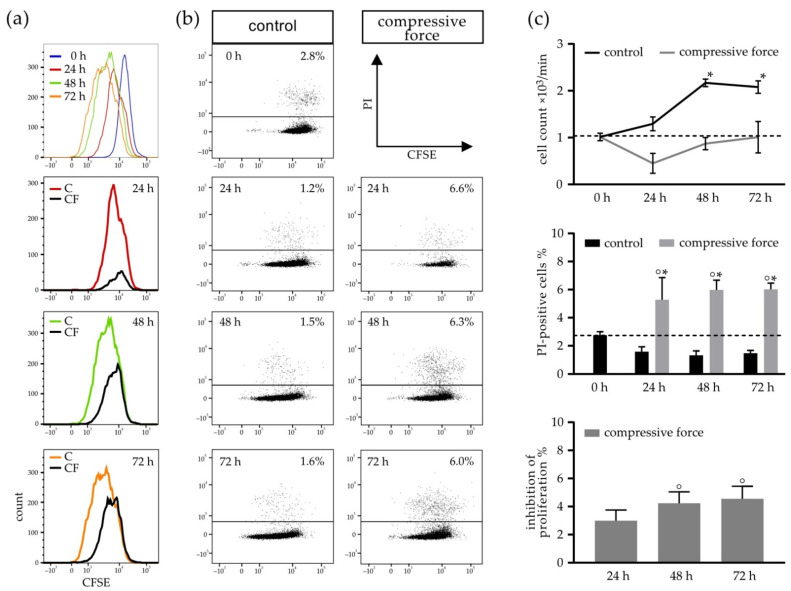
Disparities in CFSE–PI-stained hPDLFs between the control (C) and compressive force groups (CF) in terms of the cell count, PI-positive cells (dead cells) and inhibition of proliferation measured by flow cytometry. Data shown is representative for the CFSE–fluorescence intensity and inhibition of proliferation in the CF (**a**) and CFSE-positive and CFSE/PI-positive events (**b**). (**c**) Evaluation of the flow cytometry results in terms of the cell count, percentage of PI-positive cells and inhibition of proliferation. Bars indicate the mean values  ±  standard deviation. A *p*-value < 0.05 was considered statistically significant. (*) Comparison of the 24-h, 48-h and 72-h (C and CF) time points with the 0-h control. (°) Comparison of the CF with the respective control of the time point.

**Figure 4:**
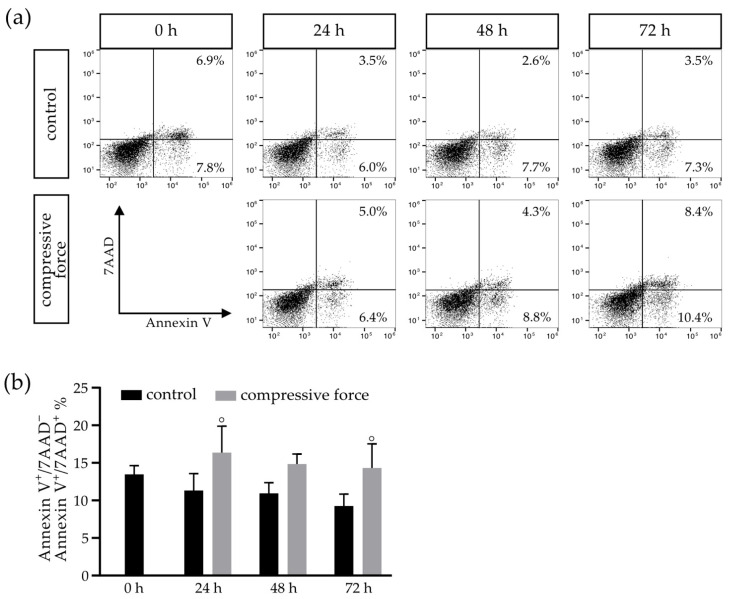
Flow cytometry examination of hPDLFs after Annexin V/7AAD staining in relation to apoptotic cell death, with an exemplary representation of all events at 0–72 h in the control (C) and compressive force (CF) groups (**a**). The total percentage of Annexin V^+^/7AAD^−^ and Annexin V^+^/7AAD^+^ events in C showed a dead cell percentage of 15%, which decreased over time, while the proportion in the CF group significantly increased (**b**). Bars indicate the mean values  ±  standard deviation. A *p*-value < 0.05 was considered statistically significant. (°) Comparison of the CF with the respective control of the time point.

**Figure 5:**
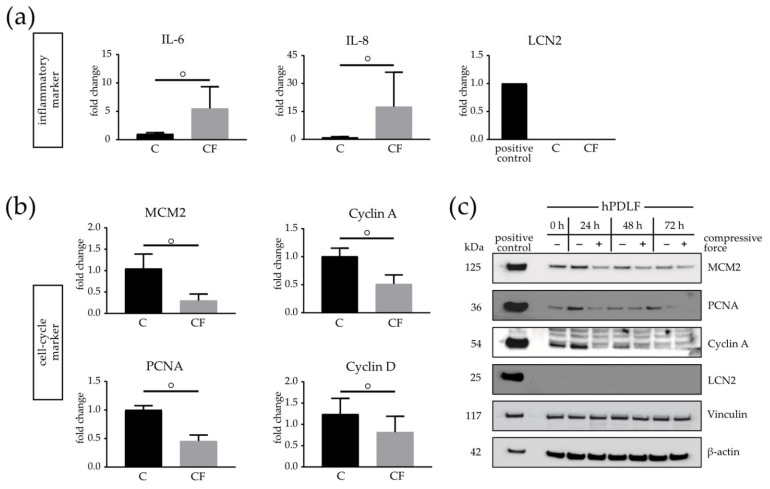
Expression of inflammatory and cell cycle markers in the presence of a compressive force (CF) compared to a control (C) group on the mRNA and protein levels. Real-time (RT) qPCR of IL-6, IL-8 and LCN2 (**a**), as well as MCM2, PCNA and Cyclin A1 (**b**), at 24 h. Cytokines were significantly upregulated in the CF group, whereas inflammatory markers were downregulated at the mRNA level. (**c**) Western blot analysis of hPDLFs from the C and CF groups at 0–72 h for the cell cycle markers (MCM2, PCNA and Cyclin A1) and inflammatory marker LCN2. β-actin and Vinculin were used as the endogenous controls. Human PC-3 cells were used as the positive control. Bars indicate the mean values  ±  standard deviation. A *p*-value < 0.05 was considered statistically significant. (°) Comparison of the CF with the respective controls of the time point.

For each method of investigation, three independent experiments were performed and measured in duplicate. Data in each graph were presented as the mean ± standard deviation (SD). First data were checked for normal distribution. Afterwards, one-way analysis of variance (ANOVA) was performed for the experiments shown in Figure 1, Figure 2, Figure 3 and Figure 4. Real-time (RT) qPCR data in Figure 5 was analyzed using the Student’s *t*-test. The statistics program was Prism version 9 (GraphPad Software, San Diego, CA, USA). A *p*-value of *p* < 0.05 was considered statistically significant. (*) Comparison to 0 h control, (°) comparison of control compressive force at the time point and (#) comparison of two different time points in the compressive force group).

## 3. Results

### 3.1. Actin Filaments of hPDLFs Changed under Compressive Force

The results show that mechanical stimulation of hPDLFs with compressive force (CF) affected the confluence and linear organization of the cells. The confluence of the control group increased continuously over the entire observation period of 72 h, and the cells appeared linearly organized (Figure 1b). In contrast, a lower density of hPDLFs was seen in the CF group compared to the respective controls. The confluence of the compressive cell group slightly increased over the 72-h observation period but remained reduced (Figure 1b). Additionally, a general disorganization of the hPDLF cell layer was noticeable. Within 72 h, the morphological changes of filamentous actin (F-actin) in the CF group were observed. An initial disorganization of F-actin was seen at the 24-h mechanical compression. After 72 h, the F-actin fibers were reinforced, and several punctual aggregates of ruptured F-actin fibers were noticed (Figure 1c).

### 3.2. Mechanical Stimulation of hPDLF Affects Cell Number and Proliferation

To further evaluate the effect of CF on the proliferation of hPDLFs, the general cell number was counted, and the amount of living and dead cells was quantified by trypan blue staining at different time points (Figure 2). In the control group, a general increase of the total cell count from the initial 2.4 × 10^4^ (0 h) up to 6 × 10^4^ cells was determined over the 72-h incubation period. Significant differences were seen at each time point compared to the 0-h control. The slope of the proliferation curve of the CF group decreased significantly after 24 h down to 1.4 × 10^4^ cells. However, after 48 h of CF, the cell number slowly increased and recovered at 72 h close to the initial value of 2.4 × 10^4^ cells of the 0-h baseline value. The increase of cell numbers in the CF group to 1.9 × 10^4^ at 48 h and 2 × 10^4^ at 72 h was significant in both cases compared to the 24-h CF. Yet, the overall reduced cell count of the CF group remains significantly reduced at each time point compared to the 0-h control (Figure 2a). Trypan blue-stained hPDLFs were considered dead. A significant rise of dead cells over time could be seen in both the control and CF groups. Especially at 48 h (4 × 10^3^ cells) and 72 h (5 × 10^3^ cells), a significant increase in CF compared to the 0-h control was measured (Figure 2b). A normalization of the number of trypan blue-positive cells to the total cell count is depicted in Figure 2c, showing a significant increase in dead cells to 25% after 24 h in the CF group. This percentage was maintained over the entire study period, whereas the percentage of dead cells of 8.5% in the control group remained unchanged over the whole observation period.

### 3.3. Compressive Force Stimulated hPDLFs Showed Inhibition of Proliferation and Reduction of Viability

To confirm the results of the manual cell count determined in trypan blue staining, the inhibition of proliferation was further investigated by the counting of living and dead cells via the flow cytometry analysis (Figure 3). An initial decrease from 1 × 10^3^ cells/min to 0.51 × 10^3^ cells/min in the CF group was seen after 24 h. It increased back to the 0-h baseline value of 1 × 10^3^ cells/min after 72 h. The slope of the control curve slightly rose after 48 h (Figure 3c, upper). However, the increases of the cell count at 48 h (2.15 × 10^3^ cells/min) and 72 h (2.05 × 10^3^ cells/min) were statistically significant when compared to the 0-h control. The percentage of PI-positive cells to the whole cell number in the CF group was significantly increased to 5% to 6% at 24, 48 and 72 h, in comparison to the 0-h control (2.4%). The juxtaposition of the CF and C groups showed significant disparities at 24 h (1.9%), 48 h (1.85%) and 72 h (1.9%), respectively (Figure 3c, middle). In addition to the reduced cell number, a significant inhibition over time of about 4% in the CF group was observed compared to the 2.5% control confidence interval at 48 h and 72 h, as shown in Figure 3c (lower).

### 3.4. Compressive Force Affects hPDLFs in a Proapoptotic Manner

To further investigate the cell death of hPDLFs under the CF kinetics of proapoptotic effects of mechanical stress over an extended incubation period of 72 h were analyzed by a flow cytometry-based apoptosis assay (Figure 4). In the C group, it was noticeable that the dead cell percentage (Annexin V^+^/7AAD^−^ and Annexin V^+^/7AAD^−^) decreased over time, from an initial 14% to 9% at 72 h, presenting an average of 12% over the 72-h observation period. Comparing the 24-h and 72-h CF groups to the 24-h (14%) and 48-h (9%) control groups, significant increases of 16% and 14.5% were observed (Figure 4b).

### 3.5. Cell Cycle and Restructuring Affected on mRNA and Protein Level in Mechanically Stimulated hPDLFs

Recently it was shown that the hPDLF response to mechanical stress is associated with an expression of inflammatory markers and enhanced cytokine production [27]. To confirm the effectiveness of the used in vitro compressive force model, several inflammatory markers were investigated by real-time (RT) qPCR analysis. In addition, it was examined whether cell cycle markers are involved in the inhibition of proliferation as a new process during the restructuration of hPDLFs under CF (Figure 5). It was found that the mRNA quantities of the inflammatory markers, including IL-6 and IL-8, were significantly increased after the 24-h compressive stimulation. Interestingly, lipocalin-2 (LCN2), an acute-phase protein inducing various cellular responses and known to be involved in extracellular remodeling, together with matrix-metalloprotease-9 (MMP-9), was not detectable in human PDLFs (Figure 5a).

The cell cycle markers PCNA, MCM2, Cyclin A1 and Cyclin D1 significantly decreased up to 50% under CF in the real-time (RT) qPCR analysis (Figure 5b). To investigate the protein levels of the respective markers mentioned above, a Western blot analysis was performed. It was observed that MCM2, PCNA and Cyclin A1 were reduced at 24, 48 and 72 h under CF in comparison to the control. However, it was found that the protein concentrations of MCM-2, PCNA and Cyclin A1 in the control group slowly approached the reduced 72-h CF group level. In line with the real-time (RT) qPCR results, no LCN2 expression on the protein levels was determinable. β-actin and Vinculin were used as the endogenous control. To demonstrate that the antibodies function properly, PC-3 cells known to express these marker proteins were used as the positive control (Figure 5c).

## 4. Discussion

OTM causes a wide-ranging reaction of the periodontium, which, through adjustment and adaptation, makes tooth movement possible without a loss of attachment. In this process, hPDLFs are considered as the maintainers of homeostasis [10,11,28]. Through the upregulation of inflammatory cytokines, information about mechanical compression and hypoxia is passed on to the surrounding tissues [12,29,30]. However, little is known about the intracellular effects of mechanical stress on PDLFs. The periodontium is a sensitive apparatus, and high compressive forces can lead to adverse effects. Therefore, in this study, a well-established moderate CF in vitro model was used, which induced sterile inflammation without having necrotic side effects [20,21]. Through this, the inter- and intracellular effects of CF can be examined. It was shown by Wu et al. that self-derived human PDLFs show a reduction of proliferation and cellular signaling to the short-term cyclic compressive strain [31].

The present study was designed to focus on the intracellular processes of CF in vitro with the aim to uncover how hPDLFs maintain self-preservation and the periodontium during early phase OTM. First, the morphological changes of the hPDLFs were examined through phalloidin/DAPI staining, as the physical connection between the cytoskeleton and the nucleus is inevitable [32]. It maintains the position of the nucleus within the cell body and provides direct mechano-transduction to the nucleus itself [33]. Other studies on fibroblasts showed that mechanical stimulation (stretch and shear stress) induced actin filament reinforcement [34,35]. In this study’s experiment, a disorganization of the F-actin fibers within the cytosol was shown. Later, the accumulation of aggregates of the ruptured fibers was seen, and a reinforcement of F-actin occurred as part of the adaptation.

Besides general cell damage due to CF exposure, including cell-disruption, previous studies revealed hypoxic damage in hPDLFs [12]. Our results indicated that the cell death rate under a moderate compressive force stayed constant after an initial increase to an average between 5–25%. In this in vitro CF model, it could not clearly be demonstrated in an apoptosis assay that the cell death mechanisms primarily regulated cell death, such as apoptosis. Yet, other studies showed that hPDLFs can mediate autophagy after an IL-1β treatment or exposure to Fusobacterium nucleatum or higher CFs (≧4 g) and react sensitive to HSP 70 inhibition [36,37,38].

Notably, this study demonstrated that mechanical stress has an impact on the proliferation of hPDLFs. They showed a marked suppression of cell cycle markers and an increased expression of inflammatory cytokines in response to CF [14,15,16,17]. IL-6 and IL-8 are known to activate acute-phase proteins and enhance remodeling of the PDLFs through recruiting lymphocytes and monocytes/macrophages [39]. LCN2, an acute-phase protein, has been found in human oral epithelial cells, serum and saliva [40,41]. Interestingly, LCN2 could not be proven to be expressed in hPDLFs. LCN2 is known to support the immune defense and extracellular reorganization through a complex formation with MMP-9, protecting it from degradation. At physiologically high concentrations of LCN2, LCN2 is thought to have a protective effect against stress [42,43]. The results of this study confirm the previous findings about an increase of proinflammatory cytokines and cell death mediators in compressive force in vitro models.

To investigate cell cycle regulation, specific markers of the G1 restriction point were used. The passage of this restriction point to the S phase activates the replication of DNA and results in cell division. The major genes and proteins of this checkpoint are cyclins and CDKs, as well as replication-associated proteins (i.e., PCNA) and MCM2. PCNA is a cofactor of DNA polymerase δ, essential for DNA replication [44]. MCM2 forms a prereplication complex with other MCM proteins for DNA replication [45]. Cyclins form complexes with CDKs and activate their kinase activity, resulting in the phosphorylation of specific transcription factors. Through the findings of this study, it is hypothesized that a lower cell division rate and a slowed cell cycle under static compression indicate that the focus in hPDLFs is to restore homeostasis of the periodontium while preserving cellular structures and simultaneously guiding the reorganization of the periodontium through increased inflammatory signaling. Thus, these cells control the transition from the early phase of tooth movement to the second phase of OTM in a protective manner.

The findings of this study gave indications for clinical in vivo situations through an in vitro experiment. This was limited by rebuilding the compression side of orthodontic tooth movement with a monoculture. Further experiments investigating the tension side will lead to further understanding of orthodontic tooth movement.

This study provided important information about the cellular response of PDLFs to mechanical stress, with a reduction of cell numbers and proliferation, and their adaptability. Our data presented novel insights into the intracellular processes in hPDLFs and static CF and helped to further understand the reorganization of PDLFs and their adaption to mechanical stress and other processes, such as hypoxia and inflammation.

Summarizing the results of this study, the initial hypotheses can partially be refuted. Human Periodontal Ligament Fibroblasts recover and adapt to the compressive force and do not generally die through mechanical compression. hPDLFs have a major influence on tissue preservation and reorganization within the periodontium during the early OTM phase.

## Figures and Tables

**Table 1:** Primer information.

Gene Symbol	Gene Name (Homo Sapiens)	5′-Forward Primer-3′ (Length/Tm/%GC)	5′-Reverse Primer-3′ (Length/Tm/%GC)
MCM2	Minichromosome maintenance complex component 2	gtggtactgctatggcggaat (21 bp/59.9 °C/52%GC)	tgagaggatcattgcctcgc (20 bp/59.4 °C/55%GC)
IL-6	Interleukin 6	catcctcgacggcatctcag (20 bp/60.32 °C/60%GC)	tcaccaggcaagtctcctca (20 bp/60.47 °C/55%GC)
IL-8	Interleukin 8	catactccaaacctttccacc (21 bp/57.9 °C/47,6%GC)	cttcaaaaacttctccacaacc (22 bp/56.9 °C/40.9%GC)
PCNA	Proliferating cell nuclear antigen	tggagaacttggaaatggaaac (22 bp/56.5 °C/40%GC)	gaactggttcattcatctctatgg (24 bp/59.3 °C/41%GC)
CCNA1	Cyclin A1	cccaagcaagggtttgacatc (21 bp/59.73 °C, 52%GC)	taccagcataggggaaactgtg (22 bp/59.76 °C/50%GC)
CCND1	Cyclin D1	gatgccaacctcctcaacga (20 bp/59.4 °C/55%GC)	gttcctcgcagacctccag (19 bp/61 °C/63%GC)
LCN2/NGAL	Lipocalin-2; Oncogene 24p3; Neutrophil gelatinase-associated lipocalin	ctccacctcagacctgatcc (20 bp/59 °C/60%GC)	acataccacttcccctggaat (21 bp/59 °C/48%GC)
RPL22	Ribosomal protein L22	tgattgcacccaccctgtag (20 bp/59.67 °C/55%GC)	ggttcccagcttttccgttc (20 bp/59.4 °C/55%GC)

Additional information about the gene function, acc. no., chromosomal location and primer location, amplicon length and location can be found in the Supplementary Data.

## Data Availability

The data underlying this article will be shared on reasonable request from the corresponding author.

## Supplementary Materials

The following are available online at https://www.mdpi.com/article/10.3390/biom11070932/s1, Table S1: Additional primer information. Figure S1: All of the Western blot materials.
