# Supplementary material for: In Vitro Compression Model for Orthodontic Tooth Movement Modulates Human Periodontal Ligament Fibroblast Proliferation, Apoptosis and Cell Cycle
Source: Biomolecules. 2021 Jun 23;11(7):932. doi: 10.3390/biom11070932 (PMC8301966; doi:10.3390/biom11070932)
Supplement: Supplementary file 1 [file biomolecules-11-00932-s001.zip › Table 1S.pdf]

Gene symbol	Gene name (homo sapiens)	Gene function	Acc. No. (NCBI Gene Bank)	Chromosomal location (length)	5'-forward primer-3' (length/Tm/%GC)	5' reverse primer-3' (length/Tm/%GC)	Primer location	Amplicon length (bp)	Amplicon location (bp of start/ stop)	Intron flanking	Variants targeted (transkript /splice)
**MCM2**	minichromosome maintenance complex component 2	initiation of eukaryotic genome replication	NM_004526.4	3; 3q21.3 (3434 bp)	gtggtactgctatggcggaat (21 bp/59.9°C/52%GC)	tgagaggatcattgcctcgc (20 bp/59.4°C/55%GC)	Exon 1/ Exon2	84	46/129	yes	yes
**IL-6**	interleukin 6	induction of transciptional inflammatory response	NM_000600	7; 7p15.3 (1127 bp)	catcctcgacggcatctcag (20 bp/60.32°C/60%GC)	tcaccaggcaagtctcctca (20 bp/60.47°C/55%GC)	Exon 2/ Exon 4	164	240/403	yes	yes
**IL-8**	interleukin 8	induction of transciptional inflammatory response	NM_000584	4; 4q13.3 (1642 bp)	catactccaaacctttccacc (21 bp/57.9°C/47,6%GC)	cttcaaaaacttctccacaacc (22 bp/56.9°C/40,9%GC)	Exon 2/ Exon 3	167	206/372	yes	yes
**PCNA**	proliferating Cell Nuclear Antigen	cofactor DNA-polymerase δ; Increase processivity of leading strand synthesis	NM_002592.2	20; 20p12.3 (1355 bp)	tggagaacttggaaatggaaac (22 bp/56.5°C/40%GC)	gaactggttcattcatctctatgg (24 bp/59.3°C/41%GC)	Exon 5/ Exon 6	95	755/849	yes	yes
**CCNA1**	cyclin A1	regulation of CDK2 and CDC2; control of cell cycle	NM_003914	13; 3q13.3 (1730 bp)	cccaagcaagggtttgacatc (21 bp/59.73°C, 52%GC)	taccagcataggggaaactgtg (22 bp/59.76°C/50%GC)	Exon 3/ Exon 4	171	515/685	yes	yes
**CCND1**	cyclin D1	regulation of CDK4 and CDK6 required for cell cycle G1/S transition	NM_053056.3	11; 11q13.3 (4238 bp)	gatgccaacctcctcaacga (20 bp/59.4°/55%GC)	gttcctcgcagacctccag (19 bp/61°C/63%GC)	Exon 1/ Exon 2	157	213/369	yes	yes
**LCN2/ NGAL**	lipocalin-2; oncogene 24p3; neutrophil gelatinase-associated lipocalin	innate immunity; maintanance of homoestasis; suppression of invasiveness and metastasis	NM_005564.5	9; 9q34.11 (820 bp)	ctccacctcagacctgatcc (20 bp/59°C/60%GC)	acataccacttcccctggaat (21 bp/59°C/48%GC)	Exon 1/ Exon 2	93	139/231	yes	yes
**RPL22**	ribosomal protein L22	component of the ribosomal 60S subunit	NM_000983	1; 1p36.31 (2061 bp)	tgattgcacccaccctgtag (20 bp/59.67°C/55%GC)	ggttcccagcttttccgttc (20 bp/59,4°C/55%GC)	Exon 2/ Exon 3	98	91/188	yes	yes
