# Supplementary figures and images for: In Vitro Compression Model for Orthodontic Tooth Movement Modulates Human Periodontal Ligament Fibroblast Proliferation, Apoptosis and Cell Cycle
Source: Biomolecules. 2021 Jun 23;11(7):932. doi: 10.3390/biom11070932 (PMC8301966; doi:10.3390/biom11070932)

Figure 5S

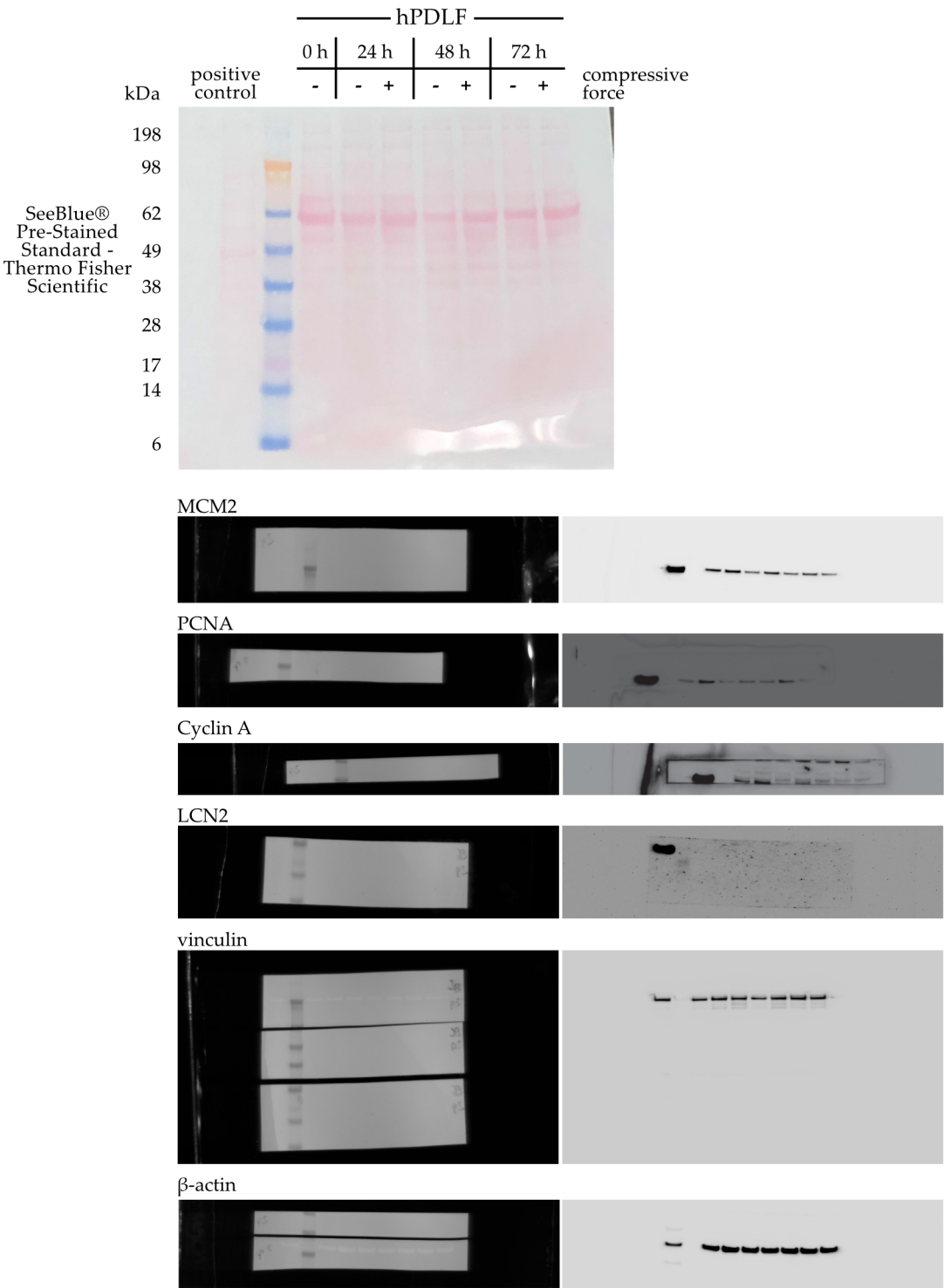

Supplement: Supplementary file 1 [file biomolecules-11-00932-s001.zip › Figure 5S.pdf]
